# Graduates in Dental Surgery for the Year 1886

**Published:** 1886-07

**Authors:** 


### EDUCATIONAL.

### Graduates in Dental Surgery for the Year 1886.

The following is the list of graduates from the Dental Colleges of the United States for the present year, except from the reports of which have not been received; also that of the "Royal College of Dental Surgeons of Ontario."

### Ohio College of Dental Surgery.

The fortieth annual commencement of the Ohio College of Dental Surgery was held at College Hall, Cincinnati, Ohio, on Wednesday evening, March 3d, 1886.

The Degree of D.D.S. was conferred on the following graduates by G. W. Keely, D.D.S., President of the Board of Trustees:

| NAME. | RESIDENCE. | NAME. | RESIDENCE. |
|---|---|---|---|
| H. L. Bryant | Minnesota. | E. G. Logan | Ohio. |
| J. C. Corcoran | Minnesota. | C. F. Materne | New York. |
| C. M. Doss | Illinois. | C. B. Meckel | Indiana. |
| C. E. Esterly | Kansas. | L. B. Moore | Ohio. |
| W. B. Gordon | Indiana. | A. F. Muenter | Germany. |
| M. H. Guthridge | Ohio. | J. Q. Neptune | Ohio. |
| W. M. Hart | Ohio. | F. T. Struckman | Ohio. |
| H. M. Howard | Ohio. | E. B. Swift | Ohio. |

R. E. Wyatt..........Ohio.

### University of Tennessee—Dental Departmnet.

The eighth annual commencement of the Dental Department of the University of Tennessee was held in connection with that of the Medical Department, in the Masonic Theatre, Nashville, Tenn., on Friday evening, February 26, 1886.

The degree of D.D.S. was conferred on the following graduates by Hon. John L. Moses, President of the Board of Trustees:

| NAME. | RESIDENCE. | NAME. | RESIDENCE. |
|---|---|---|---|
| J. S. Cottrell | Tennessee. | G. W. Myers | Kentucky. |
| E. F. Cunningham | Tennessee. | D. G. Nisbet | Mississippi. |
| C. L. DeShields | S. Carolina. | A. W. Palmer | Tennessee. |
| C. N. Harrell | Georgia. | M. L. Rudolph | Tennessee. |
| W. S. Jordan | Georgia. | J. C. Spivey | Mississippi. |
| W. D. King | Alabama. | E. G. Stuart | Texas. |

Duff Post..........Florida (honorary).

### Baltimore College.

The forty-sixth annual commencement of the Baltimore College of Dental Surgery was held at the Academy of Music, Baltimore, Md., on Saturday, March 6th, 1886.

The degree of D.D.S. was conferred on the following graduates by Prof. R. B. Winder, Dean of the Faculty:

| NAME. | RESIDENCE. |
|---|---|
| W. F. Andrews | Massachusetts. |
| Abraham L. Ashbrook | Pennsylvania. |
| George W. Baker, Jr. | Pennsylvania. |
| Harry F. Baynes | Canada. |
| August Burghard | Florida. |
| Charles W. Bradsher | North Carolina. |
| Walter F. Brown | South Carolina. |
| Emory A. Bryant | Colorado. |
| Currey Cappel | Louisiana. |
| Howard H. Carroll | Maryland. |
| Charles E. Colardeau | West Indies. |
| Henry S. Colding, M. E. | Georgia. |
| R. G. Covode | Pennsylvania |
| Wallace M. Downey | Pennsylvania. |
| Winfield B. Dulaney | Virginia. |
| James P. Farley | Massachusetts. |
| J. William Foley | Maryland. |
| J. F. Gregg | Pennsylvania. |
| J. Eayre Hendrickson | Dist. Columbia. |
| E. Hill | Georgia. |
| Bolling Hobson | Virginia. |
| Silas Hubbell | New York. |
| Sandy H. Houston, A. B. | Pennsylvania. |
| H. Herbert Johnson | Georgia. |
| Henry A. Joyner | North Carolina. |
| George Charles Keller | Maryland. |
| Charles C. Laubach | New Jersey. |
| A. C. Lindsley | New York. |
| Robert H. Marshall | Louisiana. |
| John W. Mitchell | Marylard. |
| J. Edgar Orrison | Virginia. |
| James M. Ovenshire | New York. |
| L. Earnest Payne | Maryland. |
| Albert A. Pearson | Alabama. |
| J. F. Patterson | New York. |
| Worthington Pinney | New Jersey. |
| Robert S. Russell | Tennessee. |
| Robert Owen St. Clair | Virginia. |
| Robert W. Starr | Maryland. |
| Frank W. Stiff | Virginia. |
| Aljarine T. Summerlin | Georgia. |
| Thomas C. Vankirk | Pennsylvania. |
| Wm. H. Weaver, A. B. | Georgia. |
| W. Budington Wright | New York. |

### University of Maryland—Dental Department.

The fourth annual commencement of the Dental Department of the University of Maryland was held at the Academy of Music, Baltimore, Md., on Wednesday, March 17, 1886.

The degree of D.D.S. was conferred upon the following graduates by Hon. S. Teackle Wallis, L.L.D., Provost of the University:

| NAME | RESIDENCE. |
|---|---|
| Emil Amend | Germany. |
| Frank A. Baden | Maryland. |
| Horace E. Basehore | Pennsylvania. |
| Emile Brugeille | France. |
| Thomas .W Bookhart | South Carolina. |
| William W. Bruce | West Virginia. |
| Oscar J. Campbell | Virginia. |
| Augustus H. Chafee | South Carolina. |
| John S. Diehl | Pennsylvania. |
| Joseph G. Emerson | Brazil, S. Am. |
| Charles Luff Furman | New York. |
| Elly A. Gasque | South Carolina. |
| A. H. Greenwalt, D.D.S. | Pennsylvania. |
| Charles W. Hartwig | Maryland. |
| John H. Hoffman | Virginia. |
| G. Allen Huggins | South Carolina. |
| William H. Lowell | Pennsylvania. |
| Frank H. Lumsden | Maryland. |
| Lloyd T. Macgill, Jr. | Maryland. |
| Wilfred A. Pleasants | Virginia. |
| W. Eppes Proctor, Jr. | Virginia. |
| Ralph C. Purnell | Maryland. |
| James M. Riley | North Carolina. |
| Lewis N. Shields | Texas. |
| Benjamin F. Sims | South Carolina. |
| Frank E. Slocum | New York. |
| Joseph A. Wall | Pennsylvania. |

### Pennsylvania College of Dental Surgery.

The thirtieth annual commencement exercises of Pennsylvania College of Dental Surgery were held at the Academy of Music, Philadelphia, on Saturday, Feburary 27, 1886, at 8 o'clock P. M.

The degree of D.D.S. was conierred on the following graduates by S. W. Gross, M.D., President of the Board of Trustees:

| NAME. | RESIDENCE. |
|---|---|
| J. Frank Adams, L.D.S. | Canada. |
| Theodore Balderson | Pennsylvania. |
| Franklin H. Benner | Pennsylvania. |
| William F. L. Biddell | Pennsylvania. |
| Albert C. Blind. | Pennsylvania. |
| A. P. Brubaker, A.M. M.D. | Pennsylvania. |
| Anna Castner | Germany. |
| William T. Chambers | Ohio. |
| Louise M. Diederich | Germany. |
| Henry Fraser | West Indies. |
| Benjamin H. Goodsell | New York. |
| C. Fred. Gould | Iowa. |
| William E. Holland | Pennsylvania. |
| Harry G. Keeler | New Jersey. |
| James H. Keisel | Pennsylvania. |
| Wilbur A. Kessler | Pennsylvania. |
| Henry G. Kemper | Pennsylvania. |
| Lucius A. Kelsey. | New York. |
| John P. Libhart | Pennsylvania. |
| David Souper Lyon | New Jersey. |
| Woodward R. McCloskey | Pennsylvania. |
| J. Eugene Mohr | Pennsylvania. |
| A. Milton Musser, Jr. | Utah. |
| Frank W. Monroe | Pennsylvania. |
| Johan Nawroth | Germany. |
| Olga Neymann, B.L. | New York. |
| Nettie Ogilvie | West Indies. |
| Walter J. Phillips | Iowa. |
| J. Thomp. Price | Ohio. |
| Charles S. Potts | Pennsylvania. |
| Levi Pownall | Pennsylvania. |
| J. Clark Rankin | Pennsylvania. |
| Edwin P. Robinson | New York. |
| James S. Rutter | Pennsylvania. |
| J. Herbert Sahler | Pennsylvania. |
| J. G. Santana | Cuba. |
| Maria M. Schneegans | Germany. |
| Henry C. Smale, L.D.S. | England. |
| Norberto E. Soto | U. S. of Colombia. |
| George A. Swann | Canada. |
| Charles Swap | Missouri. |
| James Tait. | Pennsylvania. |
| Oliver H. Taft | New York. |
| Thomas W. Thomas | Pennsylvania. |
| James E. Weirick | Pennsylvania. |
| Park W. Wicks | New York. |
| John J. Whaley | Canada. |
| Olga Wernickie | Pennsylvania. |

### University of Michigan College of Dental Surgery.

The eleventh annual commencement of this department was held in connection with that of the other departments of the University, on Tnursday, July 1, 1886.

The degree of D.D.S. was conferred upon the following persons:

| NAME. | RESIDENCE. |
|---|---|
| Henry Leo Banzhot | Wisconsin. |
| Edmund Keys Clements | Minnesota. |
| William Albert Courtney | Michigan. |
| Herbert Cox | New Zeland |
| Harry Williamson Davis | Kansas. |
| Henry Addison Dawley | Michigan. |
| George James Dennis | Ohio. |
| Frank Fringer Douds | Ohio. |
| Calvin Ezra Fitzgerald | Ohio. |
| Felipe Gallegos | Cent. Am. |
| Charles Perry Hanson | Michigan. |
| Anastasia Helen Hefter | Germany. |
| William A. Hoover | Ohio. |
| Ralph Hoyt | New York. |
| Michael Wallis Lau | Michigan. |
| Edwin Emery Lobb | Michigan. |
| Caroline Ada Magness | England. |
| Thomas John Mason | Indiana. |
| Arthur Henry McCann | Quebec. |
| Matilda Nehls | Michigan. |
| Charles Stillman Page | Michigan. |
| Albert Rysdorp | Michigan. |
| Clifford Francis Snyder | Wisconsin. |
| Frederick Mott Thompson | Michigan. |
| Marie A. Thompson | New York. |
| Walton Kellogg Walrath | Michigan. |
| George Henry Watson | New York. |
| Benjamin Franklin Yates | Michigan. |
| Merritt C. Hutchins | Michigan. |

### Philadelphia Dental College.

The twenty-third annual commencement exercises of the Philadelphia Dental College were held at the Academy of Music, Philadelphia, Friday, February 26, 1886, at 8 o'clock P. M.

The degree of D.D.S. was conferred on the following graduates by the President of the Board of Trustees:

| NAME. | RESIDENCE. | NAME. | RESIDENCE. |
|---|---|---|---|
| Henry Behrens, Jr | New York. | Adolph Jackson | California. |
| Elsworth E. Bentzel | Pennsylvania. | William B. Knox | New York. |
| Bernard B. Gray | Texas. | A. R. Markel | Pennsylvania. |
| Cornelia Brown | Pennsylvania. | Grant Mitchell | Ohio. |
| Gordon Brown | Missouri. | William H. Newton | Massachusetts. |
| Wm. A. Bryant (M.D.) | California. | Charles C. Patton | Maine. |
| Harry H. Burchard | Pennsylvania. | Will S. Payson | Maine. |
| Irwin G. Burton | Delaware. | Frank A. Post | New York. |
| Alonzo H. Carlile | Delaware. | Hugh G. Pullen | Canada. |
| George W. Cochran | Ohio. | Walter J. Quinlan | Canada. |
| Charles W. Collins | New York. | Charles H. Riggs | Florida. |
| W. R. G. Downes | Canada. | Whitman C. Robbins | South Africa. |
| James R. F. Fitzpatrick | Massachusetts | John P. Ruff | New York. |
| H. Edward Forrester | New York. | A. G. Smith | Ohio. |
| Arthur B. Freeman (M.D.) | Illinois. | F. W. Smith | Maine. |
| Clayton G. Gable | Pennsylvania. | Charles E. Stephenson | Pennsylvania. |
| Henry L. Gilmour, Jr. | New Jersey. | Lee K. Stewart | Illinois. |
| Jennie R. Gould | Pennsylvania. | C. E. Thompson | New York. |
| J. A. Greenawalt | Pennsylvania. | Curtis B. Tiley | Connecticut. |
| A. C. Manfred Hafstrom | Sweden. | Henry T. Walker | Texas. |
| Glyndeur Hickman | Pennsylvania. | J. Collord White | Pennsylvania. |
| W. H. J. Holman | Pennsylvania. | Seymour M. White | Pennsylvania. |
| Charles W. Huntington | New York. | W. H. White | Canada. |
| Ernest J. Husband | Canada. | Harman Yerkes | Pennsylvania. |
| Otto E. Inglis | New Jersey. | M. Pastor U. Zegers | Chili. |

### Chicago College of Dental Surgery.

The fourth annual commencement exercises of the Chicago College of Dental Surgery were held at the First Methodist Church, Chicago, on Wednesday afternoon, March 31, 1886.

The degree of D.D.S. was conferred upon the following graduates by Dr. James A. Swasey, President of the Board of Directors:

| NAME. | RESIDENCE. | NAME. | RESIDENCE. |
|---|---|---|---|
| Harry Tenn Carson | Illinois. | Joseph Perry Mertes | Wisconsin. |
| Emory Melvil Cheadle, M.D. | Oregon. | Theodore Felix Molt | Illinois. |
| Louis Chismann | Illinois. | Robert Ellsworth Moon | Indiana. |
| Joseph Grant Emery | Illinois. | Otto Henry Staehle | Illinois. |
| Gilbert Walter Entsminger | Illinois. | James Stewart | Illinois. |
| Frank Eshbaugh | Illinois. | Thomas Benton Wheeler | Illinois. |
| Ernst August Huxmann | Illinois. | Ellsworth Otis Whipple | New York. |
| Henry Frederick Marcoux | Illinois. | Alfred Rogers Wilcox | Illinois. |

### University of Pennsylvania—Department of Dentistry.

The seventh annual commencement of the Dental Department of the University of Pennsylvania was held, in connection with that of the Medical Department, at the American Academy of Music, Philadelphia, on Saturday, May 1, 1886, at 10:30 o'clock A. M.

The degree of D.D.S. was conferred upon the following members of the dental class by William Pepper, M.D., L.L D., Provost of the University:

| NAME. | RESIDENCE. |
|---|---|
| James E. Adams | Illinois. |
| A. F. Bennefeld | Germany. |
| W. A. Bordon | New Jersey. |
| C. M. Bordener | Pennsylvania. |
| W. V. Bradley | Connecticut. |
| John Campbell | Pennsylvania. |
| W. M. Chambers | Pennsylvania. |
| G. T. Cookingham | Massachusetts. |
| W. W. Danel | Illinois. |
| C. H. Davis | New Hampshire. |
| J. S. Dennison | New York. |
| Victor Dumas | Cuba. |
| C. L. Ensign | New York. |
| F. R. Griffin | Ohio. |
| W. W. Hawke | New Jersey. |
| J. B. Hills | District of Columbia. |
| F. H. Howland | Massachusetts. |
| W. S. Huber | Pennsylvania. |
| H. D. Hurlburt | Vermont. |
| L. A. Lamoutte | Porto Rico. |
| W. L. Long | Pennsylvania. |
| A. A. McIntyre | Pr. Edward's Island. |
| B. G. Maercklein | Wisconsin. |
| R. E. Maercklein | Wisconsin. |
| L. J. Miller | Pennsylvania. |
| U. S. G. Moore | Pennsylvania. |
| A. Nittinger | Pennsylvania. |
| J. Paranhos | Brazil. |
| F. Pereira | Brazil. |
| E. P. Quick | Pennsylvania. |
| O. B. Raupp | Brazil. |
| C. H. Rees | Kentucky. |
| C. H. Richter | Wisconsin. |
| F. J. Schwarzschild | California. |
| H. G. Seeley | Connecticut. |
| G. H. Shannon | New York. |
| F. I. Sumner | New York. |
| C. W. Upp | Illinois. |
| A. T. Webb | Illinois. |
| J. H. Wible | Pennsylvania. |
| L. M. Wiggins | New Brunswick. |

### Royal College of Dental Surgeons of Ontario.

The eighteenth annual examination of the students of the Royal College of Dental Surgeons of Ontario, Canada, was held in Toronto, March 2 to 5, 1886.

The following received certificates of license to practice dentistry in Ontario and the title of L.D.S.:

| NAME. | RESIDENCE. |
|---|---|
| J. G. Banerman | Brantford. |
| J. H. Carrique | Palermo. |
| C. E. Church | Ottawa. |
| A. M. Clark | Guelph. |
| J. A. Fissiault | Gananoque. |
| G. T. Fitzgerald | London. |
| Robert Haslett | Guelph. |
| R. S. Ludlow | Orangeville. |
| W. A. Leggo | Ottawa. |
| E. A. Martin | Clinton. |
| J. A. Marshall | Shelburne. |
| Charles McKinley | Georgetown. |
| James Stirton | Guelph. |
| Joseph Nolin | Montreal. |
| Ashley Weese | Napanee. |
| W. M. Wonder | St. Catherines. |

### Vanderbilt University—Department of Dentistry.

The seventh annual commencement of the Department of Dentistry of the Vanderbilt University was held in the University Chapel, Nashville, Tenn., on Wednesday evening, February 24, 1886, at 8 o'clock.

The degree of D.D.S. was conferred upon the following graduates by L. C. Garland, L.L.D., Chancellor of the University:

| NAME. | RESIDENCE. |
|---|---|
| E. W. Blakemore | Tennessee. |
| Gilbert F. Brown | Illinois. |
| A. P. Campbell, Jr. | Kentucky. |
| Thomas Cole | Georgia. |
| Wilson H. Cotton | Kentucky. |
| Thomas Crenshaw | Georgia. |
| J. S. Fann | Georgia. |
| John E. Ferguson | Georgia. |
| L. M. Frink | Florida. |
| Edgar N. Fruit | Kentucky. |
| R. M. Galloway | South Carolina. |
| Jacob W. Guerard | South Carolina. |
| Samuel J. Harrell | Louisiana. |
| Robert L. Hensley | Texas. |
| Delus Haynes | Kentucky. |
| W. B. Houston | South Carolina. |
| George A. Louque | Louisiana. |
| A. D. Lampkin | Mississippi. |
| S. H. Keener | Tennessee. |
| Frank H. McCalla | Georgia. |
| N. B. McLean | Texas. |
| George T. Neal | Georgia. |
| W. A. Patrick | Alabama. |
| S. J. Powell, Jr. | Louisiana. |
| Thomas A. Pope | Tennessee. |
| E. E. Prothro | Tennessee. |
| J. F. Ramsay | North Carolina. |
| S. C. A. Ruby | Tennessee. |
| M. S. Sale | Virginia. |
| W. F. Slaughter | Alabama. |
| John L. Stokes | South Carolina. |
| A. C. Strickland | South Carolina. |
| John W. S. Spurgeon | North Carolina. |
| Manley Timmons | South Carolina. |
| M. J. L. Townend | Mississippi. |
| Bennett Truly | Mississippi. |
| D. B. Turner | Tennessee. |
| J. E. White | Georgia. |
| William N. White | Kentucky. |
| Edwin T. Winkler | Georgia. |

### University of Iowa—Dental Department.

The fourth annual commencement of the Dental Department of the State University of Iowa was held at the Opera House, Iowa City, Iowa, on Monday evening, March 1, 1886.

The degree of D.D.S. was conferred upon the following graduates by J. L. Pickard, L.L.D., President of the Faculty:

| NAME. | RESIDENCE. |
|---|---|
| Wm. J. Brady | Iowa. |
| J. C. Allender | Iowa. |
| H. M. Baird | Iowa. |
| Geo. Babcock | Iowa. |
| E. M. Crawford | Iowa. |
| M. J. Doolittle | Illinois. |
| O. A. Dunham | Iowa. |
| W. W. Donaldson | Iowa. |
| J. E. Fleener | Iowa. |
| Geo. E. King | Iowa. |
| L. D. Hodge | Iowa. |
| B. B. Hyler | Iowa. |
| C. M. Lathrop | Iowa. |
| H. A. Leininger | Illinois. |
| T. G. Vernon | Iowa. |
| B. H. Woodward | Iowa. |
| E. G. Woodrow | Iowa. |
| B. H. Mommer | Indiana. |

### Missouri Dental College.

The twentieth annual commencement of Missouri Dental College was held in connection with that of St. Louis Medical College, at Memorial Hall, St. Louis, Mo., on Thursday evening, March 4, 1886.

The degree of D.D.S. was conferred on the following graduates by H. H. Mudd, M.D., Dean:

| NAME. | RESIDENCE. | NAME. | RESIDENCE. |
|---|---|---|---|
| A. S. Halstead | Illinois. | H. L. McKellops | Missouri. |
| W. W. Hart | Illinois. | G. L. Mock, M.D | Missouri. |
| T. E. Heatherly | Illinois. | R. Rembe | Missouri. |
| A. J. McDonald | Missouri. | Charles Summa | Missouri. |

### Indiana Dental College.

The seventh annual commencement of Indiana Dental College was held in the College Lecture Room, Indianapolis, on Wednesday evening, March 3, 1886.

The degree of D.D.S. was conferred on the following graduates by W. L. Heiskell, D.D.S., President of the Faculty:

| NAME. | RESIDENCE. | NAME. | RESIDENCE. |
|---|---|---|---|
| L. L. Clark | Massachusetts, | J. H. Palin | Indiana. |
| A. L. Jones | Indiana. | A. S. Price | Kentucky. |
| E. E. Jones | Illinois. | W. H. Rowand | Ohio. |
| O. S. Linn | Indiana. | R. M. Smiley | Indiana. |
| J. E. Montgomery | Pennsylvania. | E. E. Stewart | Ohio. |
| W. N. Wilson | Indiana. | | |

### Kansas City Dental College.

The annual commencement exercises of Kansas City Dental College were held, in connection with those of Kansas City Medical College, at Music Hall, Kansas City, Mo., Tuesday evening, March 16, 1886.

The degree of D.D.S. was conferred upon the following graduates of the dental class by Prof. E. W. Schauffler, President of the Faculty:

| NAME. | RESIDENCE. | NAME. | RESIDENCE. |
|---|---|---|---|
| J. N. Chipley | Colorado. | M. Tullis | Great Bend, Kansas. |

### New York College of Dentistry.

The twentieth annual commencement of the New York College of Dentistry was held at Chickering Hall, New York City, on Wednesday evening, March 10, 1886.

The degree of D.D.S. was conferred on the following graduates by M. Mc N. Walsh, Esq., President of the Board of Trustees:

Charles B. Atkinson.

Edward Bornschein.

Alfred Berghammer.

Henry N. Betting.

Sands J. Bowman.

Charles A. Bush.

William J. Brenan.

Victor G. Barr.

Ernest Burt.

Furman Clayton.

Joseph C. Clegg.

Willis W. Coon.

George D. Coen.

Andrew McC. Crandall.

Frank C. Chamberlain.

Elwood C. Davis.

Lewis Engle.

Edward D. Frost.

William F. Heath.

John I. Hart.

Charles De W. Henry.

Ferdinand Heindsmann, Jr.

Edward P. Jenkins.

William H. Kenzel, Jr.

Frederick B. Keppy.

George Koch.

Isaac W. Knapp.

John C. Lynch.

Edmund E. Minner.

Frank J. Maynard.

George P. Manville.

Frank A. Myrick.

Thomas W. Onderdonk.

Bissell B. Palmer.

William M. Slack.

Sidney E. E. C. K. Smith.

George J. Schreiber.

George C. Sanders.

Leverett Somers.

George Sandhusen.

Louis E. Stuart.

Peter S. T. M. Siqveland.

Friend M. Schell.

Ernest Sturridge.

Robert Stewart.

Archibald Taylor, Jr.

Thomas C. Treadwell.

Eugene S. Vogt.

Joseph S. Vinson.

James C. Whaley.

### Minnesota Hospital College—Dental Department.

The annual commencement of the Minnesota Hospital College, Dental Department, was held, in connection with that of the Medical Department, at the Hennepin-avenue M. E. Church, Minneapolis, Minn., February 26, 1886.

The degree of D.D.S. was conferred upon the following graduates by C. H. Hunter, M.D., President of the Faculty:

| NAME. | NAME. |
|---|---|
| John Dickson. | W. R. Martin. |
| C. V. R. Doolittle. | E. J. Morrison. |
| George W. Dysinger. | C. L. Remington. |
| Alger W. French. | W. H. Shaver, M.D. |

